# Epidemiology of neonatal mortality: a spatial and multilevel analysis of the 2019 mini-Ethiopian demographic and health survey data

**DOI:** 10.1186/s12887-023-03838-0

**Published:** 2023-01-17

**Authors:** Temesgen Worku Gudayu

**Affiliations:** Department of Clinical Midwifery, School of Midwifery, College of Medicine and Health Sciences, University of Gondar, Gondar, Ethiopia

**Keywords:** Neonate, Mortality, Ethiopia, Spatial, Multilevel

## Abstract

**Background:**

Sub-Saharan African countries are a high-burden region of neonatal mortality and showed slow progress in its reduction. In developing countries, as long as the current trend of mortality persists, achieving a sustainable development target for neonatal mortality would be challenging. The aim of this study was to detect significant geographic areas and identify community and individual-level predictors of neonatal mortality in Ethiopia to draw attention to a policy.

**Methods:**

A weighted total sample of 24,136 mothers from the 2019 mini-Ethiopian demographic and health survey data were included in the analysis. Global Moran’s I statistics was run to check the clustering of neonatal mortality and then kriging interpolation was done to predict the magnitude of neonatal mortality in Ethiopia. In addition, SaTScan analysis was also executed to identify hot spot clusters of neonatal mortality. Finally, a multilevel mixed-effect logistic regression model was used to identify community and individual-level predictors of early neonatal and neonatal mortality.

**Results:**

The lifetime early neonatal and neonatal mortality among mothers in Ethiopia was 5.08 (95% CI: 4.13–6.03) and 6.54 (5.55, 7.52) per 1000 births respectively. Neonatal mortality was spatially clustered in the country and the SaTScan analysis identified significant hotspot areas of neonatal mortality in the Amhara and Afar regions and some areas of the Somali and Oromia regions. Its predicted magnitude was > 8 per 1000 births in wide areas of the Amhara and Benishangul regions. A multilevel mixed-effect logistics regression analysis identified that a lower level of maternal education, being a twin neonate, and being a male neonate were predictors of both early neonatal and neonatal mortality. Whereas, the younger age of mothers predicted neonatal mortality.

**Conclusions:**

Neonatal mortality in Ethiopia is geographically clustered and sociodemographic and obstetric factors played a significant role. Policy direction should focus on evidence-based practices like midwives-led community and facility-based continuum of care from preconception to postnatal periods to possibly reduce neonatal mortality.

## Background

Neonatal mortality is a global public health concern [1] and the problem is strikingly high in developing countries. Low-and-middle income countries, particularly, sub-Saharan African countries are suffering from a high burden of neonatal mortality and showed slow progress in neonatal mortality reduction [2]. Neonatal mortality is also unequally distributed and almost 80% of newborn deaths in 2016 were from the two regions; southern Asia accounted for 39%, and similarly, sub-Saharan Africa 38% of all such deaths [3]. In Ethiopia, according to the 2016 demographic and health survey report, neonatal mortality was 29 per 1000 live births. It was only about a 24% decrease over 5 years [4]. Amhara is the leading region in the country that suffered from a huge burden of neonatal mortality. Most of the deaths during the neonatal period also unevenly occur in the first week of life [3].

Neonatal mortality, particularly early neonatal death is mainly attributed to complications of preterm birth, infection, low birth weight, and intrapartum-related events [3, 5–7]. Some large-scale studies [6, 8–10] also identified sociodemographic factors such as lack of education, obstetric factors like perinatal care none/low utilization, maternal morbidity, neonatal factors like sex, multiple gestations, gestational age at birth, and morbidity were significantly associated with neonatal mortality. At large, neonatal mortality is associated with the quality of maternal and fetal care predominantly around the time of childbirth and the death is preventable as well as treatable with well-known and cost-effective interventions [3].

Studies revealed that several interventions were found to decrease neonatal mortality. Maternal care before and during pregnancy namely, preconception care, antenatal care, prenatal nutrition counseling, and iron and multi-micro-nutrient supplementation are proven interventions. Likewise, helping babies breathe and basic resuscitation, cord care, kangaroo mother care, and exclusive breastfeeding are significant interventions to reduce neonatal mortality [11–14]. Similarly, community-level interventions [13, 15, 16] like participatory women’s groups and community health education intervention as well as improving access to high-quality antenatal and postnatal care found to decline neonatal mortality. Moreover, facility-level interventions [15, 17, 18] such as the availability of comprehensive emergency obstetrics, quality-improving interventions, and availability of maternity waiting homes are among significant factors to reduce neonatal mortality.

However, based on the current trends, 30 million deaths of newborn infants would occur between 2017 and 2030. About 80% would occur in south Asia and sub-Saharan Africa [3]. In the past 5 years in Ethiopia, no significant change in neonatal mortality was reported between 2016 and 2019 [19]. Yet studies done in the country are mainly devoted to neonates admitted in hospitals [20–22] and a small-scale communities [23]. These studies potentially lack national representativeness. In addition, other studies which analyzed the 2016 Ethiopian demographic and health survey data [8, 24, 25], didn’t consider the spatial analysis and are not timely for future planning. Thus, investigating geographic locations of a high burden of neonatal death and identifying contributing factors based on current data could be supportive of interventions to achieve the Sustainable Development Goal (SDG).

## Methods

### Study area

The mini-Ethiopian demographic and health survey (EDHS) was done in Ethiopia, a country in the Horn of East Africa. The country is of diverse geography and population. Since May 1991, the country is arranged into nine regional administrative states and two city administrations. The regions are further subdivided into 68 zones, 817 districts, and 16,253 kebeles administrative structures [4]. In the country, health service is structured into a three-tier system: primary, secondary, and tertiary levels of care [26].

### Data source and sampling procedure

This, secondary data analysis, is done by using the 2019 mini–Ethiopian Demographic and Health Survey (EDHS) data set. The sampling frame used in the survey was the census enumeration areas (EAs) created for the upcoming Ethiopian Population and Housing Census (PHC).

**Fig. 1:**
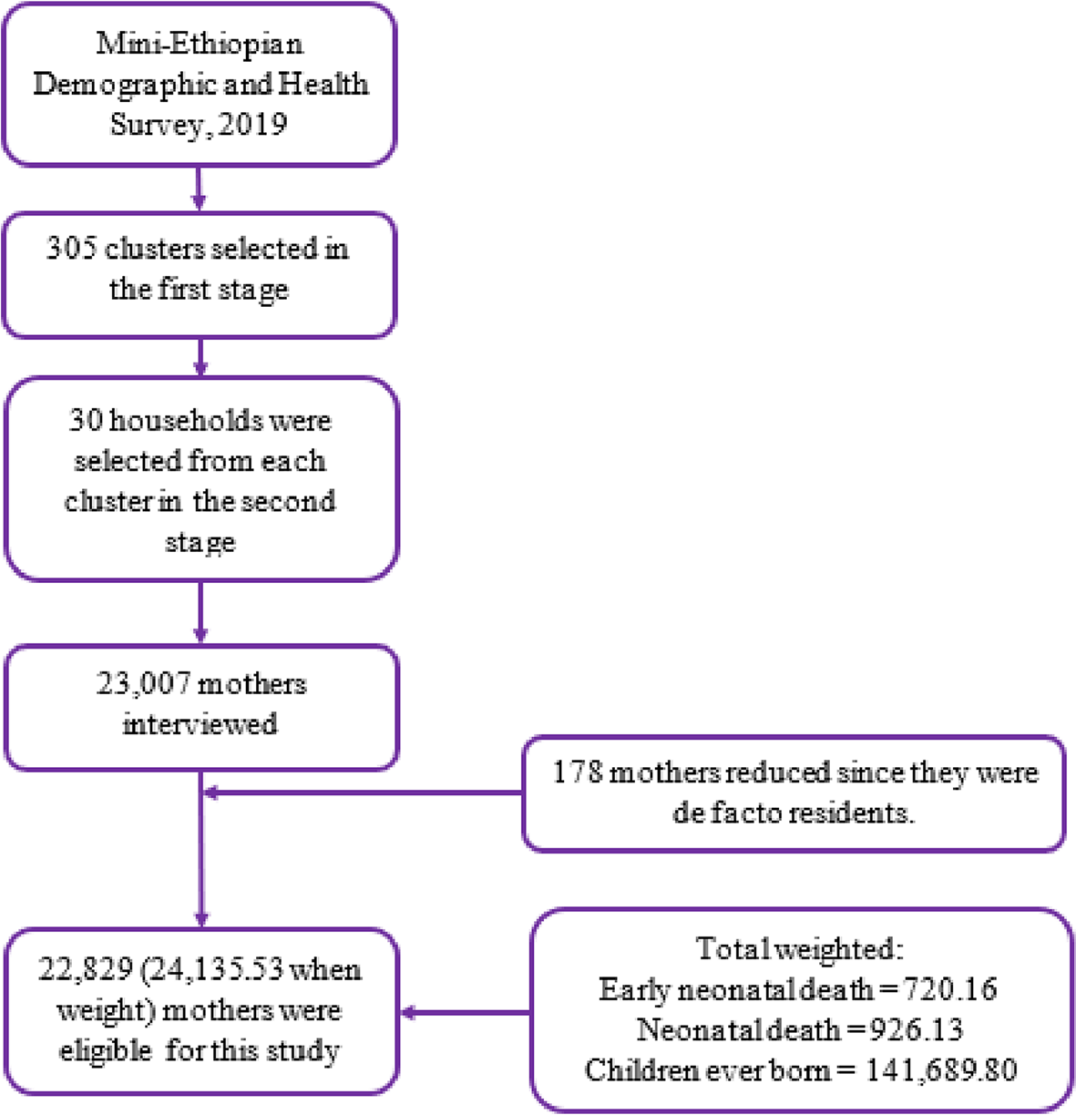
Flowchart of data extraction and sampling procedure, mini-Ethiopian demographic and health survey, 2019

The EDHS is a nationally representative two-stage cluster cross-sectional survey. As described in detail in the EDHS 2019 report [19], in the first stage, 305 EAs (93 urban and 212 rural) were selected with probability proportional to EAs size and with independent selection for each sampling stratum (urban and rural). Then, in the second stage, 30 fixed households per cluster were selected with an equal probability of systematic selection. In the current study, as shown in the figure (Fig. 1), a weighted total of 24,136 mothers in the reproductive age (15–49 year) group and a weighted neonatal and early neonatal deaths, as well as the total number of children even born from each mother were included.

### Study variables

The definition of the outcome variables of this study was made according to the World Health Organization (WHO) classification that can be accessed from https://www.who.int/data/gho/indicator-metadata-registry/imr-details/67. Accordingly, Early Neonatal Death (END) in this analysis was defined as the death of a newborn baby between zero and 7 days after birth. Whereas, Neonatal Death (ND) was defined as the death of a newborn baby between zero and 28 days after birth.

Early neonatal and neonatal deaths were measured and coded as “Yes = 1" whether the mothers ever experienced it in their lifetime and “No = 0" when they never had. In the data set the death and its corresponding date since birth was reported. From this information, END and ND were computed by dividing them by the total live births ever given by mothers and multiplying the result by 1000.

The independent variables used in this analysis were both individual and community-level variables. Maternal age, maternal education attainment, wealth index, number of babies at birth, sex of the child, and birth order were among individual-level variables. Whereas, region, place of residence, altitude of the residence, and community-level poverty were among community-level variables.

Community-level poverty was generated by aggregating individual-level variables at the community (cluster) level. The poorest and poorer family income categories were re-categorized as ‘poor’. Then, the prevalence of the new variable was divided by the cluster size, and the generated value was further categorized as ‘low’ and ‘high’ based on the median value.

### Data analysis

#### Statistical analysis

The sociodemographic and reproductive characteristics of the study participants, and the outcome variable were described in frequency and percentage. Both descriptive and regression analyses were done by Stata 14.0 statistical software.

A multilevel (cluster/enumeration areas and individual level) mixed-effect binary logistic regression model was used to analyze the association between the outcome and independent variables. Four models were used in this analysis. The first, namely, the null model (Model I) was used to check the variability of neonatal mortality in the cluster/community/enumeration areas. Model I provided evidence to calculate random effect by using an interclass correlation (ICC) whereas, the other three models, multilevel mixed-effect models, were used to identify factors associated with outcome variables. The three models were, Model II, a model comprised of individual-level independent variables, Model III, of the community-level variables, and Model IV, the final multivariable model that included both individual and community-level variables. In the last three models, independent variables that had an association with early neonatal and neonatal mortality at a *p*-value of < 0.2 during bivariate analysis were considered. In the final model, an odds ratio, its 95% confidence interval, and a p-value of < 0.05 were used to declare statistical significance.

Measures of variation (random effect) were estimated by using intraclass correlation (ICC) of ≥0.05, median odds ratio (MOR), and proportional change in the variance (PCV). The details of these estimations were discussed elsewhere in scientific articles [27–30]. Finally, models were compared based on deviance and the final which had the lowest deviance was considered the best-fitted model.

#### Spatial analysis

In the EDHS dataset, 305 clusters or EAs with their corresponding latitude and longitude coordinates were included. The Global Moran’s I statistics was done by using ArcMap 10.8 to evaluate whether the pattern of neonatal death is clustered, random, or dispersed across the study clusters. For the clustered outcome variable (based on Moran’s index *p*-value), spatial interpolation by using ArcMap 10.8 was computed to predict the burden of mortality in the country. Also, scan statistics by using a Bernoulli probability model of SaTScan V.9.6 were carried out to detect clusters and a scanning window with low or high rates of mortality. Relative risk, log-likelihood ratio, and *p*-value were reported and the statistical significance of the hot spot cluster/scanning window was declared by a *p*-value of < 0.05.

## Results

### Sociodemographic, reproductive, and neonatal mortality-related characteristics of study participants

A total of 24,136 weighted samples of mothers were included in the analysis. The age of mothers ranges from 15 to 49 years with a median age of 35 (IQR = 39–40) years. Nearly two-thirds (67.64%) of mothers had not attended formal education and only about a fiftieth (1.97%) of mothers attended secondary and above level of education. Concerning reproductive characteristics, almost two-thirds (66.05%) of mothers gave birth to five and more children (Table 1).

The weighted lifetime early neonatal mortality amongst mothers in Ethiopia was 5.08 (95% CI: 4.13–6.03) per 1000 births. Similarly, the weighted neonatal mortality was 6.54 (5.55, 7.52) per 1000 births. The mortality was higher among mothers younger than 30 years of age with a twice higher rate among teenage mothers. Also, among mothers who gave twin births and those whose birth order was one, both early neonatal and neonatal mortality were found to be high (Table 1).

**Table 1:** Sociodemographic, reproductive, and geographic characteristics of mothers in Ethiopian by early neonatal and neonatal mortality, 2019

Variables	Weighted		Mortality per 1000 births	
	Frequency	Percent	Early neonatal (95% CI)	Neonatal (95% CI)
**Maternal age**				
15–19 Years	268	1.11	36.59 (9.59, 63.59)	64.94 (23.39, 106.47)
20–24 Years	1394	5.78	14.84 (8.19, 21.48)	18.12 (9.97, 26.26)
25–29 Years	4047	16.77	10.03 (7.13, 12.93)	12.17 (9.11, 15.22)
30–34 Years	4662	19.32	5.22 (3.27, 7.16)	6.32 (4.33, 8.30)
35–39 Years	5356	22.19	3.02 (2.02, 4.01)	4.48 (3.02, 5.94)
40–44 Years	4648	19.26	4.89 (3.21, 6.58)	6.19 (4.31, 8.07)
45–49 Years	3760	15.58	3.59 (2.43, 4.76)	4.65 (3.24, 6.07)
**Maternal education attainment**				
No education	16,326	67.64	4.52 (3.58, 5.46)	6.03 (4.98, 7.08)
Incomplete primary	5974	24.75	6.69 (4.86, 8.54)	7.93 (6.01, 9.86)
Complete primary	607	2.52	7.49 (−0.19, 15.18)	7.60 (−0.10, 15.30)
Incomplete secondary	754	3.12	6.03 (0.81, 11.25)	9.67 (0.74, 18.60)
Complete secondary	131	0.54	9.48 (−4.53, 23.49)	9.48 (−4.53, 23.49)
Higher	344	1.43	5.27 (1.09, 9.45)	5.30 (1.12, 9.48)
**Wealth index**				
Poorest	5055	20.95	4.53 (3.37, 5.69)	5.91 (4.45, 7.37)
Poorer	5005	20.74	5.81 (3.99, 7.63)	7.24 (5.28, 9.21)
Middle	4982	20.64	5.18 (3.43, 6.92)	6.99 (5.15, 8.83)
Richer	5287	21.91	5.19 (3.24, 7.14)	6.57 (4.31, 8.83)
Richest	3806	15.77	4.49 (3.12, 5.88)	5.67 (3.97, 7.37)
**Number of babies at birth**				
Singleton birth	23,601	97.78	4.88 (3.97, 5.80)	6.19 (5.27, 7.12)
Twin birth	535	2.22	12.09 (6.47, 17.70)	18.49 (10.82, 26.16)
**Sex of the child**				
Male	12,486	51.73	6.30 (4.97, 7.63)	7.99 (6.65, 9.33)
Female	11,650	48.27	3.80 (2.98, 4.62)	5.01 (4.06, 5.96)
**Birth order**				
1	5801	4.30	10.19 (8.08, 12.30)	13.17 (10.81, 15.53)
2–4	5801	29.65	4.57 (3.39, 5.74)	5.94 (0.63, 7.18)
≥ 5	6745	66.05	3.41 (2.50, 4.33)	4.29 (3.37, 5.23)
**Marital relation**				
Never in union	84	0.35	17.64 (−2.46, 37.74)	17.87 (−2.24, 37.98)
Currently in union	21,978	91.06	5.06 (4.11, 5.99)	6.47 (5.44, 7.49)
Formerly in union	2074	8.59	5.09 (2.32, 7.87)	7.09 (4.37, 9.83)
**Sex of household head**				
Male	20,252	83.91	5.17 (4.16, 6.18)	6.60 (5.51, 7.69)
Female	3883	16.09	4.61 (2.76, 6.46)	6.19 (4.49, 7.88)
**Region**				
Tigray	1398	5.79	5.79 (3.42, 8.19)	7.13 (4.08, 10.18)
Afar	257	1.07	5.15 (3.76, 6.54)	6.43 (4.76, 8.09)
Amhara	4916	20.37	4.87 (3.28, 6.47)	7.69 (5.75, 9.63)
Oromia	9906	41.04	5.45 (3.84, 7.06)	6.71 (4.88, 8.55)
Somali	1413	5.86	5.57 (4.10, 7.03)	6.25 (4.56, 7.94)
Benishangul	279	1.15	8.49 (5.27, 11.73)	9.45 (6.02, 12.88)
SNNPR^a^	5255	21.77	3.98 (1.69, 6.27)	5.05 (3.35, 6.75)
Gambela	100	0.41	7.14 (3.40, 10.88)	8.21 (3.92, 12.50)
Harari	61	0.25	5.24 (4.02, 6.45)	6.80 (5.41, 8.19)
Dire Dawa	1177	0.48	4.51 (3.21, 5.81)	5.99 (4.33, 7.64)
Addis Ababa	434	1.80	6.29 (3.21, 9.08)	6.64 (3.87, 9.39)
**Place of residence**				
Urban	5706	23.64	4.62 (2.20, 7.03)	6.00 (4.15, 7.85)
Rural	18,430	76.36	5.21 (4.21, 6.21)	6.68 (5.54, 7.82)
**Community-level poverty**				
High	9951	41.23	5.24 (4.02, 6.47)	6.69 (5.17, 8.21)
Low	14,185	58.77	4.96 (3.57, 6.35)	6.42 (5.12, 7.71)
**Altitude above sea level**				
< 500 m	554	2.30	3.22 (1.74, 4.69)	3.86 (2.05, 5.68)
501–1500 m	4569	18.93	8.27 (5.94, 10.59)	9.19 (6.96, 11.42)
1501–2300 m	13,559	56.18	4.46 (3.50, 5.42)	5.79 (4.76, 6.82)
> 2300 m	5453	22.59	4.08 (2.52, 5.64)	6.40 (3.97, 8.84)

^a^Southern Nations, Nationalities, and People’s Region

### Spatial distribution of neonatal mortality

**Fig. 2:**
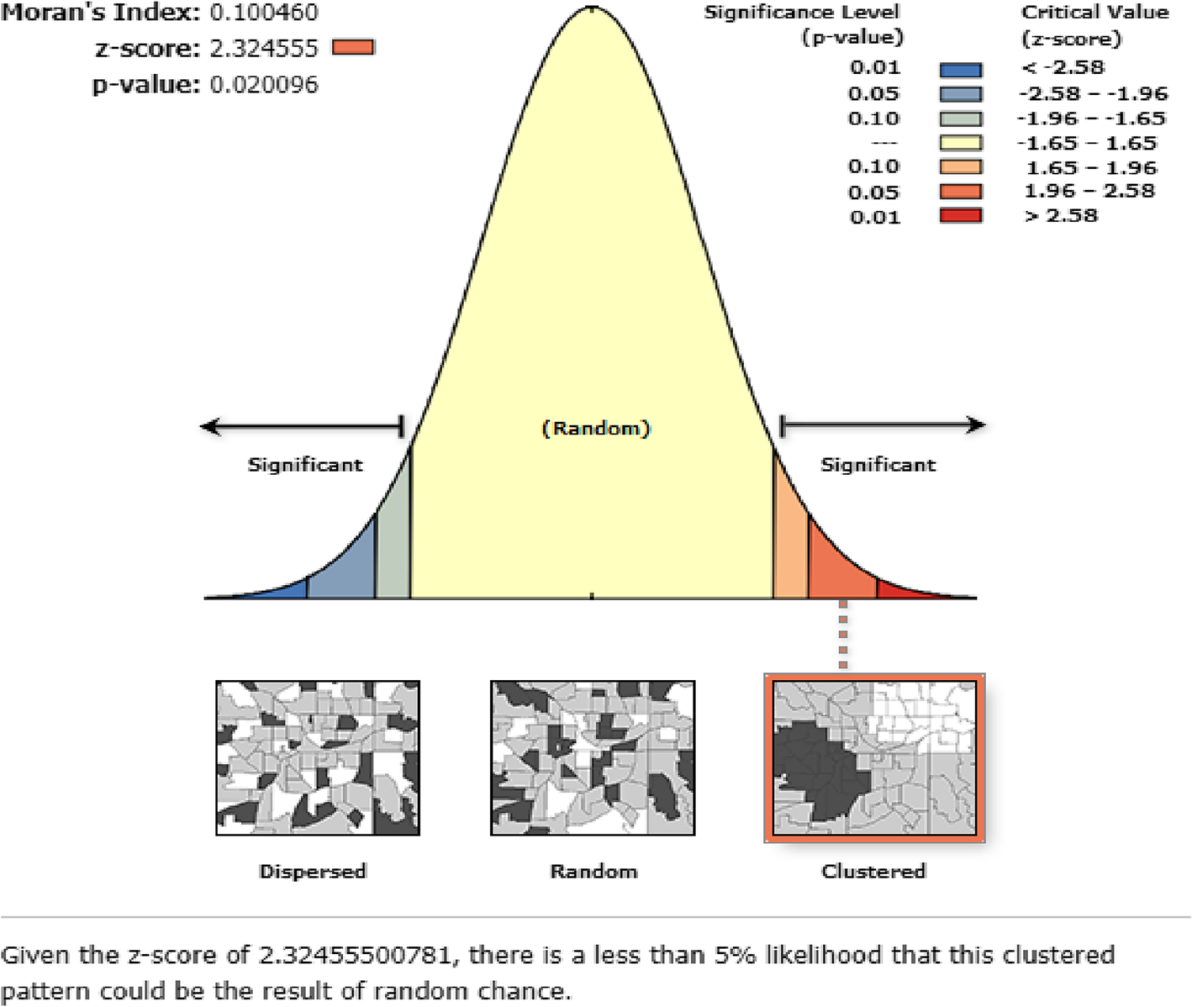
Global Moran’s I summary of spatial autocorrelation of neonatal mortality in Ethiopia, 2019

**Fig. 3:**
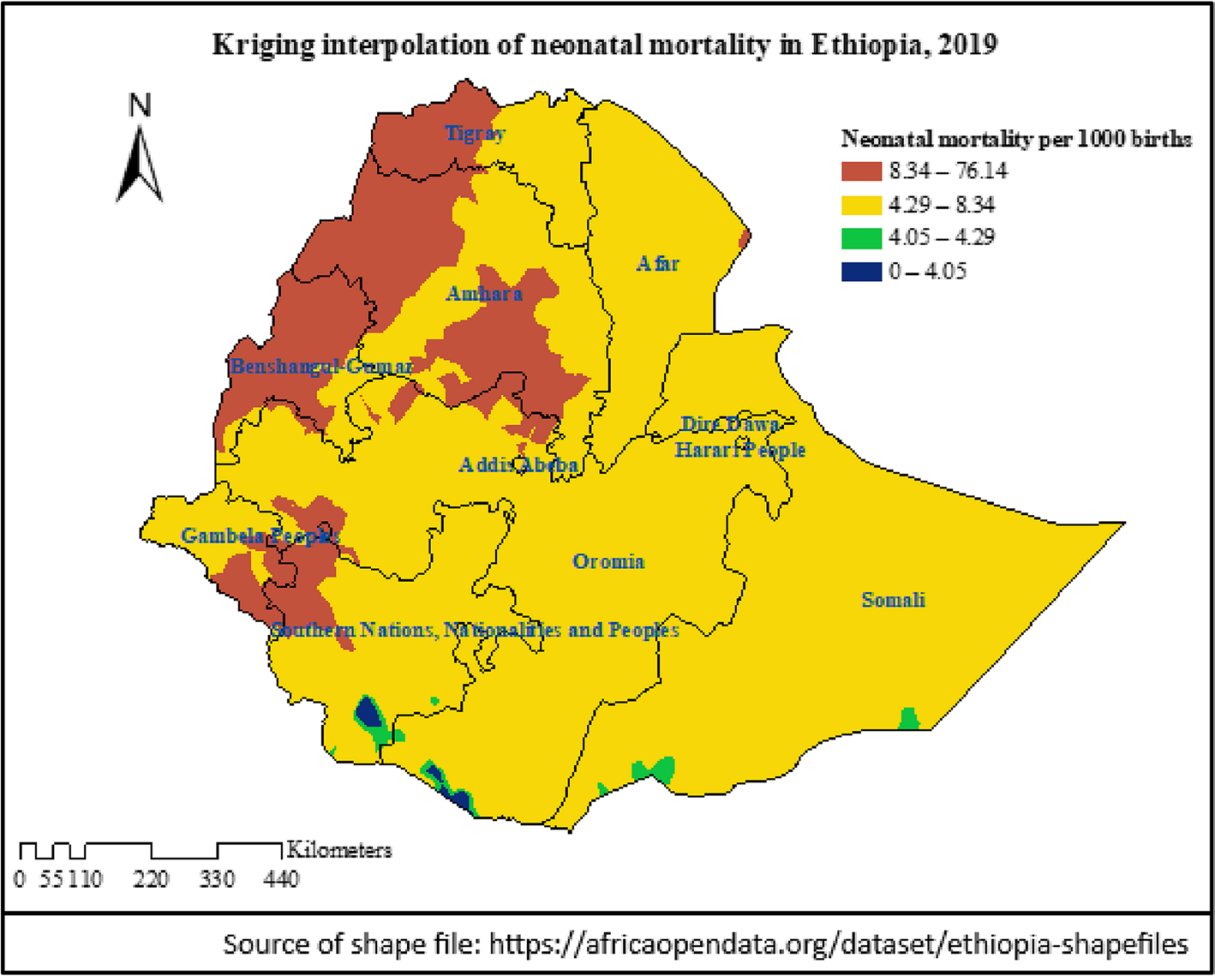
The kriging interpolation prediction of neonatal mortality per 1000 births in Ethiopia, 2019

The global spatial autocorrelation revealed a clustering pattern of neonatal mortality across the EAs (Moran’s index = 0.100460, z-score = 2.324555, *p*-value = 0.020096) (Fig. 2). In addition, kriging interpolation analysis predicted that neonatal mortality was relatively higher in northwestern, central, and southeast Amhara. Similarly, most areas of Benshangul Gumz, southern Gambela, and northwest SNNP regions had a mortality rate of > 8 per 1000 births. The neonatal mortality rate in most of the remaining parts of the country was between 4.3–8.3 per1000 births (Fig. 3).

**Fig. 4:**
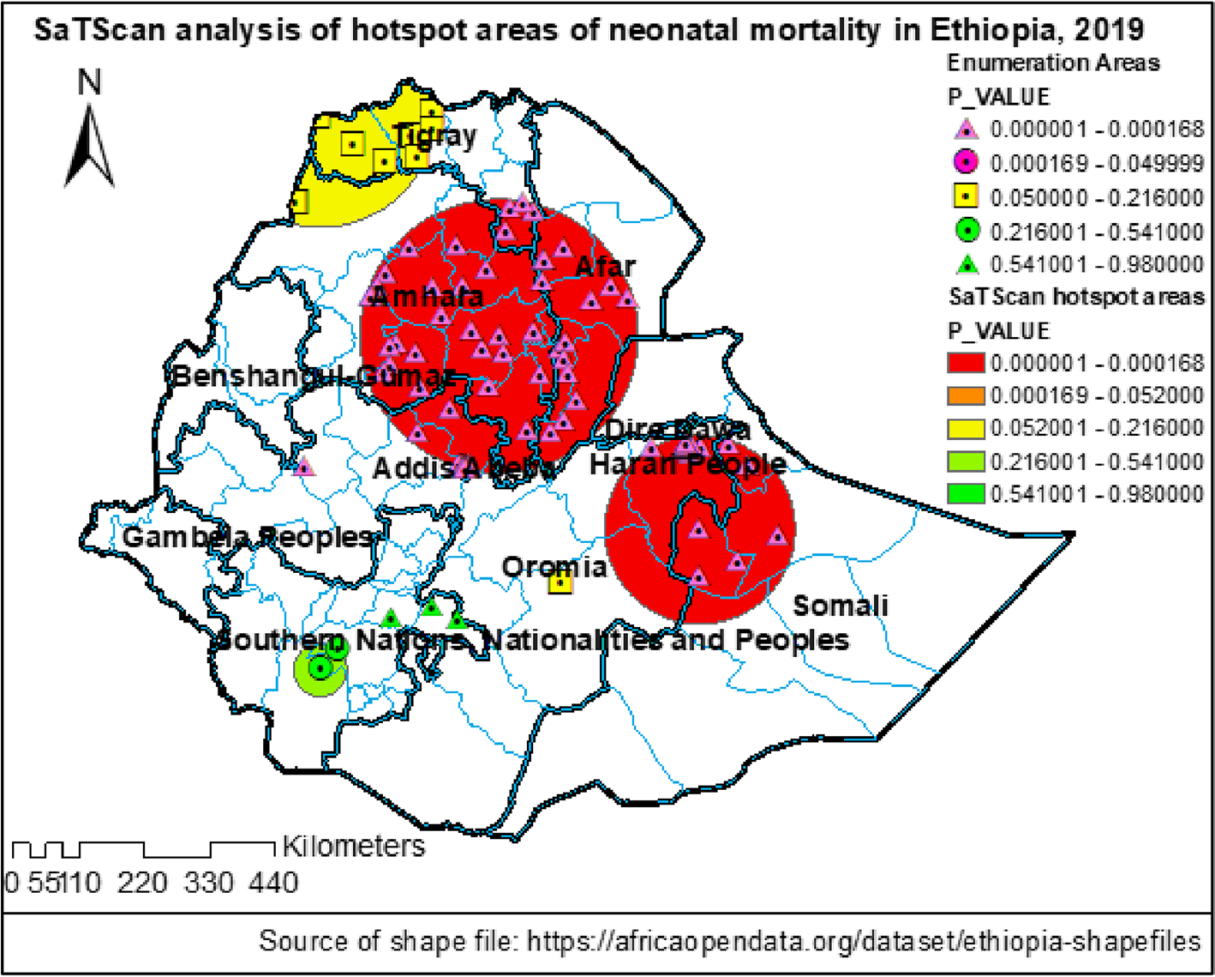
SaTScan hotspot analysis of neonatal mortality in Ethiopia, 2019

Also, a SaTScan analysis detected a total of three statistically significant cluster areas with high neonatal mortality. The most likely primary cluster area with the highest neonatal mortality was detected in the Amhara region of south Gondar, Gojjam, Wollo, Oromo, Wag Himra, north Shewa zones, and Argoba woreda. In addition, the Oromia region of west and north Shewa, Afar region of zones 1, 3, 4, and 5, and Addis Ababa City were among the primary clusters with a relative risk of (RR) = 1.67, *p*-value = < 0.001). Another most likely secondary cluster area with high neonatal mortality was spotted in the Oromia region of east Welega of Diga. The remaining secondary cluster areas were detected in the Harari region, Oromia region of east Hararge and east Bale, and the Somali region of Nogab, Jarar, Fafan, and Shabelle zones (Fig. 4, Table 2).

**Table 2:** The most likely SaTScan clusters of areas with significant neonatal mortality in Ethiopia, 2019

Location IDs^a^	Region (Zone [Woreda])	Coordinate/ radius (Km)	Relative risk	LLR	***p***-value
63, 66, 51, 65, 64, 67, 68, 60, 61, 48, 47, 62, 49, 44, 50, 58, 71, 100, 46, 29, 73, 33, 69, 78, 43, 70, 40, 18, 76, 45, 42, 72, 52, 30, 83, 99, 57, 20, 19, 59, 81, 269, 54, 31, 256, 266, 268, 257, 258, 265, 262, 271, 272, 267, 24, 263, 264, 273	**Amhara** (south Gondar, whole Gojjam, whole Wollo, Wag Himra, north Shewa, [Argoba], Oromia) **Afar** (Zone 1,3,4,5) **Oromia** (west Shewa, north Shewa) **Addis Ababa**	(10.992773 N, 39.299564 E) / 228.71 km	1.67	19.88	< 0.001
93	Oromia (East Wellega [Diga])	(9.012845 N, 36.354912 E) / 0 km	2.80	15.99	< 0.001
133, 134, 145, 250, 131, 248, 129, 249, 244, 247, 255, 107, 234, 252, 241, 245, 233, 243, 242, 235, 237, 246, 254, 239, 240, 231, 236, 232, 238, 253, 251, 121, 108	**Somali** (Nogab, Jarar, Fafan, Shabelle) **Oromia** (east Harerge, east Bale)	(8.079394 N, 42.325649 E) / 157.96 km	1.99	14.19	< 0.010

^a^Identification code of enumeration areas or clusters

### Random effect and model comparison

Random effect or community variation was assessed by ICC, MOR, and PCV. The ICC in the null model of early neonatal and neonatal mortality was 0.081 and 0.072 respectively. As indicated in the ICC result, about 8% of the variation of early neonatal mortality and 7% of neonatal mortality was attributable to the differences at the cluster level factors. The higher value of MOR (1.8) in the null model also indicated that neonatal mortality was different between clusters. In addition, the PCV value in the final model indicated that 35.75% of early neonatal mortality and 28.23% of neonatal mortality were attributable to both individual and community-level factors. Moreover, the four models were compared to select the fit model so that the final model which had the lowest deviance was considered the best-fitted model (Table 3).

**Table 3:** Random effect and model comparison for factors associated with early neonatal and neonatal mortality in Ethiopia, 2019

Parameter	Model I	Model II	Model III	Model IV
**Early neonatal death**				
ICC	0.081 (0.055, 0.117)	0.074 (0.049, 0.111)	0.057 (0.035, 0.093)	0.053 (0.031, 0.089)
PCV	Reference	8.35%	30.51%	35.75%
MOR	1.87 (1.75, 1.99)	1.82 (1.70, 1.93)	1.68 (1.58, 1.79)	1.65 (1.55, 1.75)
Model fitness	6199.10	6006.23	6162.81	5973.94
**Neonatal death**				
ICC	0.072 (0.049, 0.103)	0.066 (0.045, 0.098	0.057 (0.036, 0.087)	0.053 (0.033, 0.083)
PCV	Reference	8.24%	22.73%	28.23%
MOR	1.79 (1.69, 1.89)	1.75 (1.66, 1.85)	1.68 (1.58, 1.77)	1.64 (1.55, 1.73)
Model fitness	7299.67	7023.44	7268.02	6996.59

### Factors associated with early neonatal and neonatal mortality

Demographic and reproductive variables were analyzed in the bivariate multilevel logistic regression model. The variables which were associated with early neonatal and neonatal mortality at a *p*-value of 0.2 were further analyzed in multivariable multilevel mixed effect models (Model II and III). In the final model (Model IV), both individual-level and community-level variables were fitted to control confounders and to identify statistically significant factors of early neonatal and neonatal mortality (Tables 4 and 5).

**Table 4:** Multivariable multilevel analysis of factors associated with early neonatal mortality in Ethiopia, 2019

Variables	Model I	Model II AOR (95% CI)	Model III AOR (95% CI)	Model IV AOR (95% CI)
**Maternal age**				
15–19 Years		1.61 (0.70, 3.67)		1.59 (0.69, 3.59)
20–24 Years		1.21 (0.69, 2.13)		1.19 (0.68, 2.09)
25–29 Years		1.20 (0.80, 1.80)		1.12 (0.80, 1.79)
30–34 Years		0.94 (0.58, 1.53)		0.94 (0.58, 1.52)
35–39 Years		0.64 (0.39, 1.01)		0.64 (0.39, 1.01)
40–44 Years		1.19 (0.71, 1.99)		1.20 (0.72, 2.01)
45–49 Years		1		1
**Maternal education attainment**				
No education		2.43 (0.94, 6.30)		2.33 (0.91, 5.98)
Incomplete primary		2.68 (1.05, 6.86)		2.61 (1.03, 6.59) *
Complete primary		2.06 (0.52, 8.13)		2.03 (0.50, 8.20)
Incomplete secondary		1.32 (0.38, 4.65)		1.28 (0.37, 4.49)
Complete secondary		3.49 (0.69, 17.59)		3.58 (0.70, 18.21)
Higher		1		1
**Wealth index**				
Poorest		1.10 (0.67, 1.81)		0.95 (0.51, 1.78)
Poorer		1.41 (0.88, 2.25)		1.29 (0.74, 2.26)
Middle		1.29 (0.78, 2.14)		1.21 (0.68, 2.16)
Richer		1.47 (0.89, 2.39)		1.39 (0.70, 2.49)
Richest		1		1
**Number of babies at birth**				
Singleton birth		1		1
Twin birth		3.79 (2.48, 5.79) **		3.78 (2.47, 5.77) **
**Sex of the child**				
Male		1.65 (1.32, 2.07) **		1.65 (1.32, 2.06) **
Female		1		1
**Region**				
Tigray			1.01 (0.51, 1.99)	0.84 (0.39, 1.77)
Afar			0.83 (0.37, 1.84)	0.72 (0.31, 1.69)
Amhara			0.98 (0.53, 1.80)	0.79 (0.39, 1.60)
Oromia			1.16 (0.64, 2.12)	0.89 (0.45, 1.79)
Somali			1.26 (0.63, 2.52)	1.12 (0.50, 2.52)
Benishangul			1.47 (0.72, 3.01)	1.16 (0.51, 2.61)
SNNPR***			0.84 (0.41, 1.69)	0.63 (0.29, 1.37)
Gambela			1.68 (0.73, 3.89)	1.37 (0.55, 3.42)
Harari			1.05 (0.57, 1.94)	0.88 (0.44, 1.75)
Dire Dawa			0.67 (0.34, 1.31)	0.61 (0.29, 1.26)
Addis Ababa			1	1
**Place of residence**				
Urban			1	1
Rural			1.30 (0.93, 1.84)	1.23 (0.79, 1.89)
**Altitude above sea level**				
< 500 m			0.62 (0.33, 1.16)	0.65 (0.33, 1.26)
501–1500 m			1.54 (1.05, 2.26) *	1.49 (0.99, 2.22)
1501–2300 m			1	1
> 2300 m			0.79 (0.55, 1.16)	0.78 (0.54, 1.14)

* *p*-value < 0.001, ** *p*-value < 0.05, *** Southern Nations, Nationalities, and People’s Region

**Table 5:** Multivariable multilevel analysis of factors associated with neonatal mortality in Ethiopia, 2019

Variables	Model I	Model II AOR (95% CI)	Model III AOR (95% CI)	Model IV AOR (95% CI)
**Maternal age**				
15–19 Years		2.17 (1.04, 4.54) *		2.16 (1.04, 4.51) *
20–24 Years		1.06 (0.59, 1.89)		1.05 (0.59, 1.86)
25–29 Years		1.11 (1.76, 1.62)		1.11 (0.76, 1.62)
30–34 Years		0.86 (0.57, 1.31)		0.87 (0.57, 1.31)
35–39 Years		0.74 (0.45, 1.22)		0.75 (0.45, 1.23)
40–44 Years		1.21 (0.76, 1.93)		1.22 (0.77, 1.94)
45–49 Years		1		1
**Maternal education attainment**				
No education		3.30 (1.29, 8.48) *		3.12 (1.22, 7.94) *
Incomplete primary		3.26 (1.28, 8.32) *		3.12 (1.24, 7.87) *
Complete primary		1.91 (0.49, 7.44)		1.18 (0.46, 7.19)
Incomplete secondary		2.10 (0.57, 7.81)		2.02 (0.55, 7.43)
Complete secondary		3.39 (0.70, 16.40)		3.59 (0.74, 17.34)
Higher		1		1
**Wealth index**				
Poorest		1.16 (0.72, 1.85)		1.05 (0.57, 1.93)
Poorer		1.30 (0.84, 1.99)		1.21 (0.72, 2.03)
Middle		1.35 (0.86, 2.11)		1.27 (0.74, 2.18)
Richer		1.43 (0.89, 2.30)		1.36 (0.79, 2.34)
Richest		1		1
**Number of babies at birth**				
Singleton birth		1		1
Twin birth		4.99 (3.31, 7.52) **		5.01 (3.32, 7.56) **
**Sex of the child**				
Male		1.59 (1.31, 1.93) **		1.59 (1.31, 1.93) **
Female		1		1
**Birth order**		0.94 (0.99, 0.99) *		0.94 (0.89, 0.99) *
**Region**				
Tigray			1.37 (0.70, 2.66)	1.13 (0.55, 2.31)
Afar			1.25 (0.59, 2.67)	1.03 (0.45, 2.34)
Amhara			1.69 (0.98, 2.93)	1.35 (0.71, 2.56)
Oromia			1.57 (0.89, 2.79)	1.24 (0.64, 2.39)
Somali			1.64 (0.87, 3.12)	1.39 (0.66, 2.93)
Benishangul			1.95 (1.02, 3.75) *	1.55 (0.73, 3.28)
SNNPR***			1.17 (0.65, 2.13)	0.89 (0.46, 1.75)
Gambela			2.23 (1.02, 4.88) *	1.83 (0.78, 4.28)
Harari			1.54 (0.88, 2.69)	1.33 (0.73, 2.44)
Dire Dawa			1.07 (0.57, 2.01)	0.96 (0.49, 1.87)
Addis Ababa			1	1
**Place of residence**				
Urban			1	1
Rural			1.21 (0.91, 1.60)	1.11 (0.77, 1.61)
**Altitude above sea level**				
< 500 m			0.62 (0.34, 1.14)	0.64 (0.35, 1.19)
501–1500 m			1.41 (1.01, 1.98) *	1.36 (0.96, 1.93)
1501–2300 m			1	1
> 2300 m			0.94 (0.65, 1.35)	1.93 (0.65, 1.34)

* *p*-value < 0.001, ** *p*-value < 0.05, *** Southern Nations, Nationalities, and People’s Region

Accordingly, the final model revealed that mothers whose current age was 15–19 years were about twofold [Adjusted odds ratio (AOR) = 2.16, 95% CI: 1.04, 4.51] more likely to have neonatal mortality than elder-aged mothers. Also, mothers who didn’t complete primary education were likely to experience both early neonatal and neonatal mortality. Not completing primary education among mothers resulted in above two and three times more likely mortality of early neonates (AOR = 2.61, 95% CI: 1.03, 6.59) and neonates (AOR = 3.12, 95% CI: 1.22, 7.94) than educated mothers respectively. Likewise, mothers who didn’t attend formal education were nearly three times more likely to have neonatal mortality than educated mothers (AOR = 3.12, 95% CI: 1.24, 7.87) (Tables 4 and 5).

Early neonatal and neonatal mortality was also attributed to neonatal factors. Mothers who gave birth to twin babies had a higher than a threefold chance of early neonatal mortality (AOR = 3.79, 95% CI: 2.48, 5.79), while about five times more likely mortality of neonates (AOR = 5.01, 95% CI: 3.32, 7.56). Similarly, mothers who gave birth to male neonates had a higher chance of both early neonatal and neonatal mortality than female neonates (Tables 4 and 5).

Community-level variables didn’t show a statistically significant association for both neonatal and early neonatal mortality in the final model, Model IV. However, mothers who live in a specific altitude area were more likely to have both early neonatal and neonatal mortality in Model III. As compared to middle or temperate altitudes, mothers who live in an area of lowland altitude had a greater likelihood of early neonatal (AOR = 1.54, 95% CI: 1.05, 2.26) and neonatal (AOR = 1.41, 95% CI: 1.01, 1.98) mortality (Tables 4 and 5).

## Discussion

Neonatal mortality is a worldwide public health issue and one of the leading health concerns in low-and-middle-income countries [1, 3, 31]. This study detected significant hotspot areas and identified community and individual-level factors that contributed to neonatal and early neonatal mortality in Ethiopia.

Neonatal mortality in Ethiopia is geographically clustered in wide areas of the Amhara and Afar regions and some areas of the Somali region and eastern zones of the Oromia region. Similarly, a higher geographic variation of neonatal mortality in the 2016 EDHS was reported and it was revealed that the rate was high in Oromia and Afar regions next to Amhara and Somali regions [4]. Neonatal mortality was found to be predicted by maternal education attainment, less antenatal attendance [32, 33], adolescent pregnancy [33], and home births [5]. This attribution could be informative for the aforementioned regions and areas of high neonatal mortality in Ethiopia. As indicated in reports, in the clusters of high neonatal death, more than 70% of mothers were not accessing any form of media (> 80% in the Amhara region), more than 80% of mothers didn’t complete elementary education, > 65% didn’t attend at least four visits of antenatal care, > 70% gave birth at home, and only < 30% of births were assisted by skilled attendants [4]. Also, in the Amhara region, most neonates die in the perinatal period due to prolonged labor and its complication, ruptured uterus [34]. Though neonatal mortality in Ethiopia showed a 41% reduction from 49/1000 livebirths in 2000 to 29/1000 livebirths in 2016, the regional distribution has remained higher in Afar, Amhara, Oromia, and Somali regions with the persistently highest rate (47/1000 live births) in the Amhara region [4].

In addition, low birth weight, neonatal morbidity, and maternal morbidity were found to be predictors of neonatal mortality in Ethiopia [35, 36]. Maternal nutrition and morbidities are also directly associated with low birth weight among newborn babies [37–40] and nutritional intervention is recommended to avert low birth weight thereby neonatal mortality [41–43]. In Ethiopia, studies revealed that pregnant mothers are suffering from malnutrition due to inadequate food diversity and household food insecurity [44, 45], and these further result in low birth weight and preterm birth [40, 46, 47] as well as directly in neonatal mortality [48]. Food insecurity is common in Ethiopia as revealed by studies [49–51] and significant food insecurity was observed in lowland and highland areas than in midland areas [51].

Neonatal death is statistically significant among teenage mothers in Ethiopia. Mothers whose age ranges between 15 and 19 years were about four times and those between 25 and 29 years were nearly two times more likely to have neonatal death than elderly mothers. Studies [52–58] also revealed an association between maternal age and neonatal death. There is evidence that teenagers during pregnancy face many obstetric complications like anemia, urinary tract infection, pregnancy-induced hypertension, and preterm births [59]. Among the common obstetric complications that teenagers experience, preterm birth alone increases the risk of neonatal mortality in this age group of mothers [58]. Higher neonatal mortality among younger-age mothers in this study could reflect that developing countries are still having higher neonatal mortality in the late Millenium Development Goals (MDG) and Sustainable Development Goals (SDG) era and might continue to have it in the future [3].

Mothers who didn’t attend formal education and those who didn’t complete primary education had close to three times more likely risk of early neonatal and neonatal mortality in Ethiopia. Maternal level of educational attainment was also found a predictor of neonatal death in other studies [56, 60]. In Ethiopia, female education could be affected by the deep-rooted practice of early marriage, which in turn is associated with lower education and economic dependence [61, 62]. Educated and employed mothers are more empowered to decide using maternal and childcare services [63]. It is suggested that educating girls and accessing maternal health services are among the key interventions to reduce neonatal mortality [64, 65].

Neonatal and early neonatal mortality is higher among mothers who gave male birth than female birth. Death among males was found to be one and half times higher in Ethiopia. Several studies conducted so far in Ethiopia similarly identified higher mortality among male neonates [23, 66, 67]. Similarly, as supported by other studies [67–69] done in sub-Saharan Africa, mothers who gave twin birth had about four and more times higher likelihood of death than singleton births in this study. Higher death amongst twin births could be related to significantly higher preterm birth and anomaly, and lower Apgar and birth weight [70]. Whereas, though the sex variation of mortality is inconclusive, some studies explored more susceptibility to infection, prematurity, and poor perinatal conditions in male neonates than in females [71–74].

Although the government of Ethiopia executed different health-related goals of the Millennium Development Goal (MDG) and still implementing the Sustainable Development Goal (SDG), neonatal mortality in the country remained high [19]. In the health sector transformation plan [75], Ethiopia was proposed to achieve over 90% coverage of 4+ antenatal care, deliveries attended by skilled health professionals, antiretroviral therapy for HIV-positive pregnant mothers, and postnatal care to reduce neonatal mortality to the level of 10/1000 live births in 2020. The proposed time to reduce neonatal mortality was passed, yet the Ethiopian neonatal mortality estimate for 2019 showed 27/1000 live births [76]. As revealed by some studies, among the individual interventions believed to improve neonatal health, such as health facility construction campaigns [77] and institutional delivery [78] contributed a little. Countries like Ethiopia should adapt an integrated evidence-based continuum of care approaches than a fragmented individual intervention. Therefore, as revealed and suggested by a recent Live Saved Tools Modeling study to estimate the number of deaths that would be prevented by 2035, countries that account for the vast majority of the world’s maternal and neonatal deaths and stillbirths can potentially avert the problem substantially by installing midwife-led sexual, reproductive, maternal, newborn, and adolescent interventions [79].

In this analysis, large data was used and statistically significant clusters of neonatal mortality in Ethiopia were identified. However, this analysis is mainly focused on determining the lifetime experience of neonatal mortality among mothers in Ethiopia. To understand the current magnitude and determinates of neonatal mortality, analyzing the data from a recent 5 years period would be helpful.

## Conclusion

Neonatal mortality in Ethiopia is common and actionable socio-demographic and obstetric factors were found to predict it. Also, it is geographically clustered and Amhara and Afar regions are widely affected areas in the country. Policymakers should focus on evidence-based recommendations such as; midwives-led community and facility-based continuum of care from preconception through prenatal, intranatal, postnatal, and neonatal periods to possibly reduce neonatal mortality.

## Data Availability

The datasets generated and/or analyzed during the current study are available in the DHS program repository, [https://dhsprogram.com/] upon registration with no requirement of accession number.

## Abbreviations

DHS: Demographic and Health Survey
EAs: Enumeration Areas
EDHS: Ethiopian Demographic and Health Survey
END: Early Neonatal Death
ICC: Intraclass Correlation Coefficients
LL: Log Likely
MMR: Maternal Mortality Ratio
ND: Neonatal Death
NGO: Non-Governmental Organizations
PHC: Population and Housing Census
